# Scoping review of brucellosis in Cameroon: Where do we stand, and where are we going?

**DOI:** 10.1371/journal.pone.0239854

**Published:** 2020-09-28

**Authors:** Christopher G. Laine, Abel Wade, H. Morgan Scott, Rosina C. Krecek, Angela M. Arenas-Gamboa

**Affiliations:** 1 Department of Veterinary Pathobiology, College of Veterinary Medicine & Biomedical Sciences, Texas A&M University, College Station, Texas, United States of America; 2 National Veterinary Laboratory, Ministry of Livestock, Fisheries and Animal Industries, Yaoundé, Republic of Cameroon; East Carolina University Brody School of Medicine, UNITED STATES

## Abstract

Brucellosis is a zoonotic disease known to be endemic to parts of western and sub-Saharan Africa. However, the epidemiology for humans and animals remains largely unknown in many of these countries with Cameroon being a typical example. Despite common knowledge that brucellosis affects livestock, the actual number of infected animals remains unknown. Through a scoping review, the current known status of the disease is described. The aim is to ascertain relevant and publicly accessible research and knowledge of human and animal brucellosis in the country, and to provide an overview of the factors associated with its known persistence. Seroprevalence has been estimated and published in 12 separate instances (1 human; 9 cattle; 1 human and cattle; and 1 that includes cattle, pigs, and small ruminants), between 1982 and 2020, in 9 of the country’s 10 geopolitical regions. In 1983, *Brucella abortus* and *B*. *melitensis* were isolated in cattle, but no further bacterial isolation has been published since. The seroprevalence from 196 total humans has ranged between 5.6% and 28.1%, and between 3.0% and 30.8% for 14,044 total cattle. As there is no ongoing surveillance program, it is not currently possible to identify the specific *Brucella* spp. that are endemic to the country and its regions. There are sufficient agricultural systems of cattle, pigs, goats, and sheep to sustain the presence of multiple *Brucella* spp. Surveillance information is the cornerstone of epidemiologic decision making, and is needed to direct policy makers, public health authorities, and veterinary services to appropriate actions. A combination of serological and molecular based diagnostics for surveillance is necessary to identify, quantify, and direct the appropriate public health interventions. Cameroon has an opportunity to build public and animal health infrastructure, leading the way for central Africa in the management and future eradication of brucellosis.

## Introduction

Brucellosis is a zoonotic disease known to be endemic to parts of western and sub-Saharan Africa [1–3]. This disease is caused by gram-negative bacteria from the genus *Brucella*. Currently, there are three zoonotic *Brucella* species that are highly virulent to both their respective hosts as well as to humans (i.e., *Brucella abortus*: cattle, *Brucella melitensis*: sheep and goats, and *Brucella suis*: swine) [1, 4, 5]. The nature and extent of the disease remains largely unknown in many African countries with the Republic of Cameroon being a typical example [6].

Brucellosis is a disease of significant importance in livestock. Infection can cause abortion and infertility, a decrease in milk production, as well as weak offspring, all noteworthy as causing both short- and long-term losses to agricultural economies [1, 5]. Normal transmission of the bacteria occurs during ingestion of infected milk, or contact with infected uterine secretions, fetal membranes and/or aborted fetuses [5, 7]. Maintained both in wildlife and in agricultural systems, the bacterium can also be transmitted between the systems during customary agricultural operations, such as pastoral transhumance and comingling of livestock with wildlife [6, 8].

Zoonotic brucellosis is associated with significant morbidity characterized by fever, fatigue, sweats, and malaise. In some cases, arthritis, endocarditis, and neurological disorders may occur [5, 9]. Consumption of unpasteurized milk products, and handling of contaminated tissues such as aborted placentas without adequate protection, may lead to disease transmission to humans [1, 5]. Therefore, animal handlers, abattoir workers, and veterinarians are considered to be at a higher risk for acquiring the disease [1, 5]. Disease in humans typically manifests as undulant fever with influenza-like symptoms, and can lead, in some instances, to the development of arthropathies, cardiomyopathies, neurological events, and adverse pregnancy outcomes [10].

This disease is known to be endemic in many countries throughout the African continent [2, 3]. However, in Cameroon, despite common knowledge that brucellosis affects cattle, the actual number of infected animals and the main animal species affected by the disease in the country both remain unknown. This present study describes the current known status of the disease, and the aim of the review is to ascertain relevant and publicly accessible research and knowledge of human and animal disease in the country, and to provide an overview of the factors associated with its known persistence. By better recognizing this information along with the human capacity strengths, opportunities, and weaknesses, policymakers will better be able to make informed, evidence-based decisions to reduce the disease burden in both animals and humans.

## Methods

**Fig 1:**
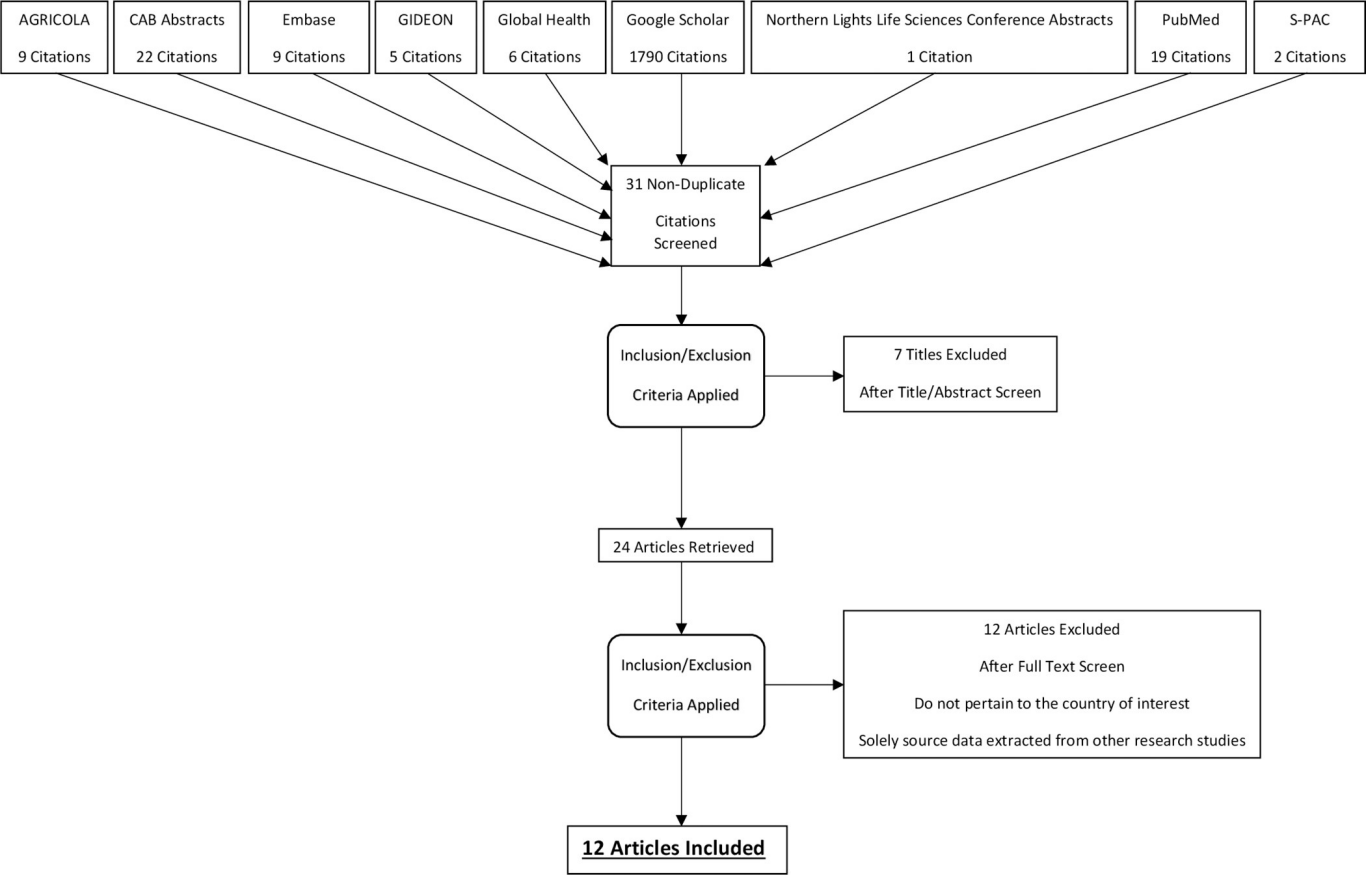
PRISMA flow diagram of the seroprevalence studies identified, screened and assessed, and included in the scoping review.

A literature search, adhering to the PRISMA guidelines [11] and following the PRISMA-ScR Checklist, was conducted in multiple databases (AGRICOLA, CAB Abstracts, Embase, GIDEON, Global Health, Google Scholar, Northern Light Life Sciences Conference Abstracts, PubMed, and S-PAC) (Fig 1). Initially, Google Scholar was searched by the criteria: brucellosis & Cameroon and *Brucella* & Cameroon. Subsequently, the database was searched by the criteria: brucellosis +Cameroon file:.pdf and *Brucella* +Cameroon file:.pdf, and then to account for French publications, *Brucella* +Cameroun file:.pdf, brucelloses +Cameroun file:.pdf, *Brucella* & Cameroun, and brucelloses & Cameroun. To account for publication bias, grey literature was considered. Based on these search terms, 31 publications were identified as relevant and non-duplicate by their title, and screened for content. No other relevant novel research was identified while searching the aforementioned databases that was not already supplied by Google Scholar. The reference sections of each article were then searched for relevant documents within the scope of the study, and nothing novel was identified. There were no restrictions on language, and research scientists at the Laboratoire National Vétérinaire (LANAVET) aided the extraction of French language literature.

There were no restrictions on the type of study or year of publication. Considering the limited number of filtered articles, broad inclusion criteria (measures of seroprevalence, epidemiology and control, and associated risk factors, as well as pastoralism and management, slaughter, dairy, and occupational health and safety practices) were utilized for the search. Articles were specifically excluded when they (i) did not pertain to the country of interest and/or (ii) solely sourced data extracted from other research studies. After undergoing a title and abstract screen, followed by a full text screen for the inclusion and exclusion criteria, 12 articles remained for inclusion. Due to the limited amount of published literature available, a review protocol was not obtained. The data was extracted independently with no need to calibrate forms, and the data was added directly to the tables.

For further understanding of the disease situation in this country, other determinants of animal and human health were considered. These included economic, geographic, and ecological aspects. Several websites were sourced for this information, including the World Organization for Animal Health, the United States Centers for Disease Control and Prevention, and the United States Central Intelligence Agency: World Fact Book. The 2016 national livestock appraisal conducted by the Cameroon Ministry of Livestock, Fisheries and Animal Industries (MINEPIA) was also sourced for this information. Additionally, a publication on the country’s animal trade network was sourced for information on animal movement [12].

### The Republic of Cameroon and livestock systems

**Fig 2:**
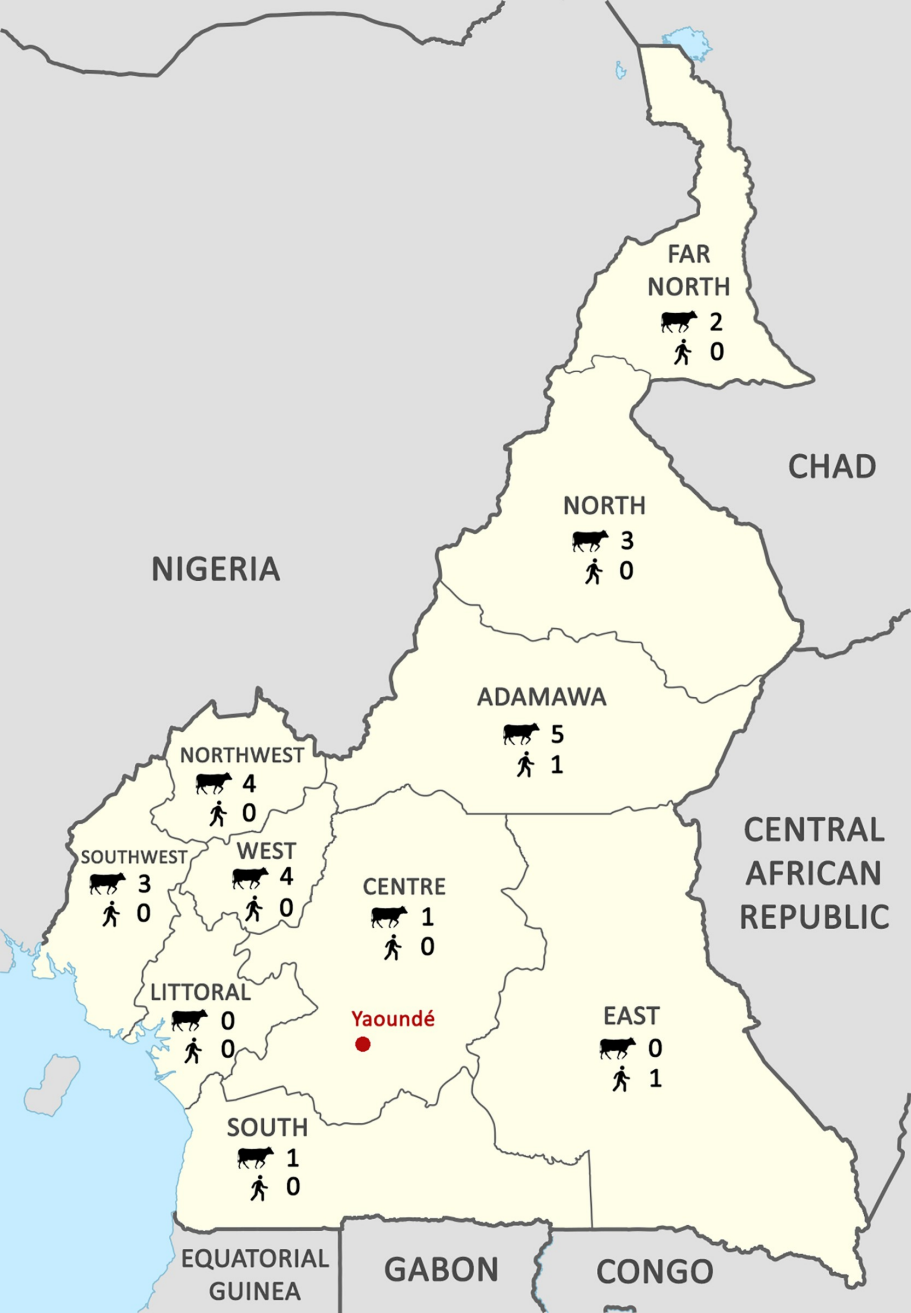
Distribution of cattle and human seroprevalence studies conducted, by region, in Cameroon. Counts are based on Tables 1 and 2. Based on UN map: Provinces of Cameroon EN.svg.

The Republic of Cameroon is located in the west of central Africa. It borders the Bight of Biafra (Atlantic Ocean) in the west, the Federal Republic of Nigeria in the northwest, the Republic of Chad at the northeast, the Central African Republic in the east, the Republic of the Congo at the southeast, and both the Republic of Equatorial Guinea and the Gabonese Republic in the south (Fig 2). The country has a geopolitical landscape consisting of ten regions (Adamawa, Centre, East, Far-North, Littoral, North, North-West, South, South-West, and West). A coastal plain is located in the cardinal southwest, a dissected plateau in the center, mountains in the west, and high plains in the north [13]. The climate also varies with each region and terrain, ranging from tropical along the coast to semiarid and hot in the north [13]. Livestock are generally raised in the northern semi-arid regions and traded in the southern forested regions along with urbanized areas [12]. The national and regional borders of the country are porous to livestock movements, allowing for mostly unrestricted intranational and international trade accompanied by disease transmission [12].

Livestock accounts for 13% of the agricultural GDP, and employs or supports 30% of the rural population [14]. One third of households operate in the livestock sector directly affected by this disease, partitioned between goats 55.1%, sheep 27.2%, pork 23.3%, and cattle (beef & dairy) 17.9% [14]. According to the 2014 MINEPIA report, there were approximately 6.3 million goats, 5.8 million cattle, 3.1 million pigs, and 3 million sheep at the time of the census [14]. For these animals, three main categories of livestock production systems co-exist, including: pastoral (extensive small and large ruminants), sedentary (extensive/semi-intensive, mixed crop-livestock), and commercial (semi-intensive) [14]. These livestock operations are dominated by approximately 95% smallholders, and the cattle, pigs, and small ruminants are a source of cash income, nutrition, food security, and social standing for households in rural communities [14].

The movements and trade of livestock are stable throughout the country, but these systems are poorly understood [12]. These systems are very much interlinked in the integrated farming systems, but are not centralized or fully regulated [12]. The main livestock producing regions are Adamawa, North-West, and West [12]. Adamawa has an official cattle population of approximately 1.25 million head and is the main source for the rest of the country [12]. Adamawa also serves as a hub for receiving animals from Chad and Central African Republic, grazing, and further transport to other regions [12]. The North-West and West Regions are also known for cattle production, but have a significantly smaller population of 450 thousand and 160 thousand respectively [12]. The porous borders allow for unrestricted animal movements between neighboring countries, and common managerial practices include transhumance of herds across regional and national boundaries [12]. As a result of extensive agriculture and transhumance, the animal ecosystem interface is characterized by interaction with other herds and wild animals, such as buffalo [12, 15].

## Results and discussion

### Seroprevalence and study characteristics

Twelve published seroprevalence studies have been conducted in the country between 1982 and 2020. The literature is wide-ranging in scope, including epidemiology and control, associated risk factors, and measures of seroprevalence, as well as pastoralism and management, slaughter, dairy, and occupational health and safety practices. The studies are summarized in Tables 1 and 2 by year of publication, region, target population tested, production system, diagnostic tests used, calculated seroprevalence, and sample size.

**Table 1:** Characteristics for published livestock seroprevalence studies conducted in Cameroon.

Year	National Region	Target Type	Production System	Diagnostic Test(s)	Seroprevalence	Sample Size	Reference
1982	Adamawa, North, and Far-North	Beef	Extensive	RBT	30.80%	7665	[16]
1993	North & Far-North	Beef	Extensive	RBT, SATT, CFT	8.40%	607	[17]
2005	West	Beef	Extensive	RBT, CFT, SAW-EDTA, iELISA	9.64%	840	[18]
2009	Western Highlands	Dairy	Dairy	C-ELISA	8.40%	192	[19]
2010	Adamawa	Beef	Banked sera	C-ELISA	3.00%	1377	[20]
2015	North-West	Beef	Extensive & Semi-intensive	C-ELISA	5.20%	689	[21]
2016	North-West	Beef	Extensive & Semi-intensive	C-ELISA	4.04%	198	[22]
2016	Western Highlands and the Guinea Highlands	Beef	Extensive & Semi-intensive	RBT	4.61%	(N/A)	[23]
2018	North & Adamawa	Beef	Extensive & Semi-intensive	RBT, iELISA	5.40%	1031	[24]
2018	Adamawa	Beef	Extensive & Semi-intensive	RBT, iELISA	3.40%	590	[25]
2020	Central, South, South-West, and West	Beef, Swine, Sheep, Goat, and Dog	Extensive & Semi-intensive	RBT, iELISA	6.35%	1873	[26]

Not available in the literature (N/A), Rose Bengal test (RBT), serum agglutination tube test (SATT), complement fixation test (CFT), slow agglutination of Wright with ethylenediamine tetraacetic acid (SAW-EDTA), indirect enzyme linked immunosorbent assay (iELISA), competitive enzyme-linked immunosorbent assay (C-ELISA)

**Table 2:** Study characteristics for human seroprevalence publications conducted in Cameroon.

Year	National Region	Target Type	Production System	Diagnostic Tests	Seroprevalence	Sample Size	Reference
2016	East	Abattoir Workers	Abattoir	RBT, iELISA	28.10%	89	[27]
2018	Adamawa	Abattoir Workers	Abattoir	RBT, iELISA	5.60%	107	[25]

Across the studies, the locations and production systems have been heterogeneous. Nine of the ten regions have been assessed (Adamawa 6, Center 1, East 1, Far-North 2, North 3, North-West 4, South 1, South-West 3, and West 4) (Fig 2). One study is referred to as the Guinea Highlands, which is located in the Adamawa region. Another study reviewed the Western Highlands, which includes the West, North-West, and South-West regions. These studies incorporated the major cattle production regions [12]. Between 1982 and 2018, investigations were split between four semi-intensive and seven pastoralist communities. Three of these studies were conducted in abattoirs, one at a dairy farm, and one with banked sera, while all other investigations were conducted at beef farms. Of these, nine of the studies assessed only cattle, one evaluated only human subjects, and one investigated both cattle and humans. Most recently, for the first time in this country, disease prevalence in small ruminants, swine, and dogs has been investigated [26]. This research was split between semi-intensive and pastoralist communities [26]. Unfortunately, the study grouped all of the different animal species together, from all of the dispersed geographic locations (tropical forest, mountains, and central plateau), in its final seroprevalence estimate, making it difficult to analyze and draw conclusions from the data [26]. Despite wildlife also being an important factor for this country, due to transhumance and extensive production systems, no studies have ever been conducted in these species.

Adequate and careful interpretation of the serological test results reported in these studies is crucial when trying to draw any significant conclusions of the current local situation. As illustrated, Tables 1 and 2 list significant wide ranges in seropositivity from 3.0–30.8%. However, it is important to highlight the discrepancy in the usage of the standard serological testing approach between studies and the interpretation of such. Represented in Table 1, five of the studies used the Rose Bengal Test (RBT) in combination with the indirect enzyme-linked immunosorbent assay (iELISA) [18, 24–27], and one used RBT in combination with the complement fixation test (CFT) for confirmation [17]. In contrast, two studies simply employed the RBT [16, 23], and the remaining four relied solely on the competitive ELISA (C-ELISA) [19–22]. Each of these tests embodies a distinctive method of identification and should be interpreted carefully as the positive vs. negative values between various tests differ. But most importantly to note is that despite the test of choice, all diagnostic tests require strict quality control and assurance through test validation for the values to be reliable. For example, despite the proven record of the utility and effectiveness in aiding in the diagnosis of brucellosis in many countries, the RBT and CFT, require an individual to determine presence of agglutination or lysis respectively which in the absence of proper antigen quality, antigen standardization, and technical training results can be inaccurate or misinterpreted. RBT is an economically good choice for resource limited settings and its use should be encouraged, but antigen quality, standardization, and test interpretation are all key aspects for accuracy [5]. As the sensitivity (Se) and specificity (Sp) of RBT has been shown to be highly variable between manufacturers over time, the tests should be interpreted only by trained personnel and antigen selection should be carefully conducted to avoid inconsistencies. Therefore, adequate standardization protocols and methods to meet the standards for test validity must be fostered. Other traditionally employed tests, such as ELISAs, display quantitative numerical results, making interpretation more standardized. However, these tests have the inconvenience of needing a threshold value for positive vs. negative determined through analysis which to the author’s knowledge has never been performed in Cameroon [4, 5]. Such tests require equipped laboratories as well as data analysis capability, to be able to identify true positives and negatives as well as to identify the statistical cutpoint at which samples with unknown disease status may be diagnostically classified as positive or negative [4, 5]. The threshold values can change along with the biological diversity between locations, so they must be recalibrated between animal species and new locations, which requires a considerable amount of time and monetary resources. In addition to the basic understanding of individual assay performance, one must recognize that a diverse range of diagnostic tests can be employed with several different approaches, and that the interpretation of the results will change based upon the approach. Assays can be used individually, or they can be combined in series or parallel strategies [4, 5, 28]. Series strategies operate through screening and confirmation, where the sample is considered positive only if it tests positive on two or more assays [29]. Parallel strategies exercise a complimentary method, where the sample is considered positive if it tests positive on any assay when two or more are employed [29]. Series interpretation increases Sp and decreases Se, as the strategy is exclusive [29]. Parallel interpretation increases Se and decreases Sp, as the approach is inclusive [29]. It is important to note that there is not a single test or strategy that is universally appropriate [4, 5, 28].

At the beginning stages of prevention and control, the World Organization for Animal Health (OIE), Food and Agriculture Organization of the United Nations (FAO), and World Health Organization (WHO) indicate that the main unit of reference for an animal surveillance program is the herd [5]. The previously published serosurveys have not consistently addressed herd prevalence adequately over the years and across all of the areas investigated. Recently, between 2010 and 2018, herd seroprevalence was calculated to be between 16% and 25.6% in Adamawa and the North regions, respectively [20, 23, 24]. However, because of improper use of diagnostic testing and interpretation, the confidence of this data is questionable. It is important to develop strong capacity and surveillance systems, so that future investigations and surveillance may properly incorporate herd prevalence into the framework.

In order to have trusted, reliable data, future studies should be conducted with the appropriate scientific rigor and approach, utilizing the current methodology for the proper use and interpretation of diagnostic testing for animals. The recommended methodology is described by the WHO, FAO, and OIE in the manual *Brucellosis in Humans and Animals* [5]. Further information can be found in the OIE *Manual of Diagnostic Tests and Vaccines for Terrestrial Animals 2019*, and the FAO manual *Guidelines for Coordinated Human and Animal Brucellosis Surveillance* [4, 28]. The most current misuse of diagnostic testing uses the same assay and combines samples from different animal species situated in geographically diverse regions. It is well known that despite the usefulness of serology for detecting antibodies against *Brucella* spp., when conducted properly and interpreted correctly, several shortcomings are noteworthy from the serological assays per se, including the probability of cross reactivity with other bacteria, such as *Yersinia enterocolitica* O:9, *Salmonella* Urbana group N, *Vibrio cholerae*, *Francisella tularensis*, *Escherichia coli* O157, and *Stenotrophomonas maltophilia*, among others [30] and the inability to differentiate between the smooth strains. Further limitations to these present studies include that in all of these investigations, smooth strain *Brucella* spp. antibodies were detected, and further bacteriological isolation and typing was only completed in 1983 to determine the species [31]. Although no further microbiological or molecular characterizations have been completed in 37 years, and while in total only 14,044 cattle samples have been examined, the limited data suggest that *Brucella* spp. is endemic in the country, indicating a need for more robust research and surveillance.

### Current status and management of brucellosis

In 1983, investigators isolated and then biotyped *Brucella* spp. using standard biochemical tests in cattle [31]. They reported isolating *B*. *melitensis* in the Far-North and *B*. *abortus* in the Far-North, North, and Adamawa regions [31]. Small ruminants are the target for *B*. *melitensis* and represent a core agricultural staple for people living in resource limited regions [6, 14, 32]. These animals are an important aspect of the greater agricultural economic system; however, only most recently has information become available in regards to the disease status.

In addition, relying solely on serological testing for screening and subsequent confirmation or complementation is not useful for distinguishing *Brucella* spp. Inclusion of the polymerase chain reaction (PCR) to compliment serology may increase testing specificity and facilitate species identification [30]. Adopting both serological and molecular based surveillance may aid in a safe and more efficient detection of *Brucella* spp. The use of molecular characterization may not be currently viable in resource limited settings, but it should be a goal for all countries attempting to implement or bolster brucellosis surveillance and control. However, it is important to mention that the availability of diagnostic capacity alone is not sufficient for an adequate surveillance system. An educated and trained veterinary workforce is required for implementation of the program and tracking of animals.

Disease maintenance and spread plays a major role in the continued propagation of brucellosis in the country and central Africa in general [8, 12]. Interestingly, one contributing factor to this might be that described in 2013 by Profitos et al, where pastoralists in the Far-North region tended to retain chronically sick animals in their herds for extended periods, even though diseases like brucellosis have persistent and negative impacts on the health of the herd [8]. In the Western Highlands, it has been shown that approximately 70% of the cattle sold at abattoirs were on the verge of death [21]. Although antibiotics (of unknown quality) are readily available, treatment is a long and expensive process for brucellosis, and if traditional medicine is believed to suffice, farmers will opt for the traditional route [8]. It is important to note that the use of antibiotics in animals for brucellosis treatment is discouraged. In addition, pastoralists have been shown to not properly employ antibiotics per the requisite course of treatment, opting instead for a “wait and see” approach, giving animals a low dosage and waiting to see changes before giving them another dose [8]. These studies show that pastoralists tried to avoid selling their sick animals, as the weight yield was low and their morbid animals had lower market value [8, 21]. Sale of diseased animals generally only occurred when death was imminent as farmers would hold off on selling animals in hope they would recover and thereby prevent further economic losses [8]. Profitos et al also showed that pastoralists tended to be more aware and concerned about other diseases like trypanosomiasis and foot-and-mouth-disease. A large percentage of farmers (89.5% in the same investigation) were not aware of brucellosis [8]. Amongst some of the risk assessments conducted in the country, knowledge and concern for brucellosis by farmers has shown to be possibly associated with disease prevalence [21, 24]. Awareness and knowledge regarding the disease and its transmission are important factors in prevention and control.

Cameroon has an ecology that includes many species of wildlife that may contract and spread brucellosis, including buffalo [15]. Nothing is known about the status of brucellosis in these species such that sylvatic maintenance of this disease cannot be understood and correlated with transmission to livestock. The agricultural practices of many farmers place their livestock in direct contact with a wide variety of ecosystems. One study indicated that wild buffalo regularly comingle with ruminant livestock [15]. These buffalo are common in certain regions, and their interaction with cattle may be an indicator for increased *Brucella* spp. seroprevalence in herds [15]. Identifying and controlling wildlife carriers may aid in reducing transmission to livestock, especially if livestock comingle during grazing.

### Public health relevance

Of the nearly 24 million people that live in Cameroon, the labor force consists of approximately 10 million, and 70% of those individuals are classified as agricultural workers [13]. This is important as brucellosis remains one of the neglected diseases in humans, exemplified by the fact that the disease is rarely diagnosed in humans and despite the overwhelming evidence of its prevalence in at-risk agricultural workers [25, 27]. Unfortunately, only 196 human serum samples have been collected for the purposes of seroprevalence research despite the apparent high seroprevalence in cattle [25, 27]. An alarming human seropositivity of 5.60% and 28.10% was detected in these two study populations. Unfortunately, these studies did not follow the guidelines for diagnostic testing and interpretation for humans, drawing into question the significance of the data [25, 27]. The proper use and interpretation of diagnostic testing for humans can be found in the WHO manual, *Brucellosis in Humans and Animals*, [5] and it should be noted that the prevention and control of brucellosis should revolve around occupational exposures and ingestion of contaminated animal products [5]. It is important to note that public health education, community participation, and training of health workers has been shown to be the backbone of human disease prevention and should be included in any future plans for disease control and prevention [5].

Exposure to the zoonotic *Brucella* spp. is a very important occupational hazard to the working population of this country [25, 27]. Abattoir workers in Cameroon are generally exposed to the full range of animal species that carry *Brucella* spp. [25, 27]. Additionally, as expected, it was observed that brucellosis is transmitted to humans during the customary and routine consumption of raw milk and animal agriculture operations in the country [25, 27]. Exposure within the abattoir is likely related to failure to employ personal and collective protective equipment [25, 27]. Unfortunately, the only two reported study conclusions have been based on serological assays without the use of bacterial culture to confirm their findings and do not indicate the source of infection [25, 27]. Despite its public health significance and the diverse exposures common throughout the population, there is no surveillance system, and it is imperative that a strategy be developed to address this deficit.

### Current challenges

As public health and economic prosperity is highly correlated with animal disease prevalence, prevention and control of this disease in animals should be a goal in resource limited settings to help scaffold this prosperity [5]. For prevention and control efforts to begin, there needs to be a high level of intersectoral collaboration between veterinary services, public health, and medical services [5]. These efforts will require the entire community to be involved and support the effort including individual citizens and local organizations, as well as each echelon ranging between local leadership and national governance [5]. Additionally, for this intersectoral collaboration to thrive, communication through education, training, data sharing, and open dialogue between stakeholders is key. Enlightening stakeholders of the negative effects of the disease as well as the benefits of surveillance and control are very important to the sustainability of such programs. In resource limited settings, the lack of funding, shortage of veterinary and public health workers, inadequate communication, and remoteness of farmers rank highly amongst the many barriers to spreading knowledge about brucellosis [33]. Furthermore, in Cameroon, decisions from MINEPIA and other ministry’s policy makers must translate to proper actions by animal science and veterinary centers (CZV), health professionals, and farmers. Under the direction of MINEPIA, there is a structure of Regional Delegations followed by Divisional Delegations, Sub-divisional Delegations, with CSV being at the ground level. MINEPIA makes policy, CZV conduct veterinary health services and collects data, and the hierarchical delegations between them act as the administrative structure. For surveillance and control to be successful, there needs to be coordination between each of these levels. A breakdown in communication at any level will compromise the entire system.

In addition to the development of solid communication networks, it is necessary to structure a sustainable surveillance program that efficiently reports accurate, relevant, and useful information. Due to the lack of public and animal health infrastructure, most parts of Africa have insufficient reporting of disease at territorial or international scales [33]. The scarcity of veterinary services, along with the inaccessibility of remote and transhumant animals, also restricts the capability of surveillance and control programs to establish a census, track animals through tagging, control animal movement, and collect data, as well as most other efforts to reduce the impact of disease [33]. In regions where data is successfully collected, the information has been known to not be communicated adequately between authorities, and these authorities generally tend to be more focused on policies controlling acutely fatal diseases [33]. It is not only important that programs are capable of properly identifying disease, but the data needs cultivation through aggregation and analysis, and the appropriate authorities are needed that are capable and willing to assess the situation as well as commit scarce resources to efficient and efficacious policies.

Insufficient funding is a major restrictive determinant of surveillance and control programs [33]. Control strategies based on eradication through test-slaughter are not realistic in resource limited settings, due to the inability to financially compensate farmers for culled animals [34]. Culling of non-infected animals is a risk due to imperfect sensitivity and specificity of diagnostic testing [33]. A proactive vaccination approach is more realistic, but still requires financial resources for veterinary services and surveillance, and requires the tracking of animals [5]. Surveillance also requires secure and sustainable access to quality reagents and materials for conducting tests. For example, the RBT reagents need to be properly standardized, optimized, and implemented to achieve proper repeatability and reproducibility of test results [4]. Economic sustainability should always be considered before investing resources into prevention and control.

### Limitations

The scoping review study design has several limitations. It was not possible to quantitatively evaluate the quality of previous studies. It was also a requirement to amass information from many different study questions, designs, and methods, over a wide range in years to provide a proper overview of the literature. In the majority of the studies completed in Cameroon, to date, the scientific rigor of data is questionable due to an inappropriate study design and/or the inadequate use and interpretation of serological assays. Due to the limited amount, and heterogeneous nature of the literature, a scoping review is the most suitable design for this study.

## Conclusion

It is important to note that the seroprevalence of brucellosis has been estimated on twelve separate instances, between 1982 and 2020, and each of these was conducted in a different location and production system; that is to say, no ongoing surveillance program exists, and thus, it is not currently possible to identify the specific *Brucella* spp. that are endemic to each of these study regions. In 1983, *B*. *abortus and B*. *melitensis* had both been isolated [31], and sufficient agricultural systems of cattle, goats, and sheep remain to sustain the presence of both *Brucella* spp. [12, 14]. It also seems that there are adequate agricultural systems for the presence of *B*. *suis*, [14]. In 2020, small ruminants and pigs were shown to be infected with a smooth strain *Brucella* spp., but the specific species remains unknown [26]. It is clear that there is a disease burden, but a continuous surveillance system utilizing standard diagnostic techniques is necessary to adequately define the current status of disease in both animals and humans. A better way forward may be a combination of serological, microbiological and molecular based diagnostics for surveillance. PCR diagnostics would allow one to distinguish among *Brucella* spp. [30]. Surveillance would then identify, quantify, and direct the appropriate public health interventions [35].

It is important to note that there is no existing animal vaccination program in the country. While this fact may be relevant in the future it is currently a moot point due to the fact that there is no effective surveillance system. In addition to needing sufficient veterinary services for a vaccination program, a surveillance program must first be instituted which constitutes the strongest component to managing the disease [28, 35]. The FAO *Guidelines for coordinated human and animal surveillance* provides details of the basic requirements for such programs [28].

Surveillance also plays a role in supplying information for the education of stakeholders. Information about infected populations and the scale of disease burden is the cornerstone of epidemiologic decision making, directing policy makers and public health authorities to appropriate actions [35]. The information is also useful for enhancing the awareness and knowledge of livestock owners about brucellosis, empowering them to make properly informed decisions about their holdings. Most importantly, the proper management of brucellosis in livestock represents the most efficient approach to decrease the disease burden in humans [36, 37]. To this end, Cameroon has an opportunity to build public and animal health infrastructure by building veterinary services capacity, instituting an effective surveillance program, educating stakeholders, and establishing efficient preventive measures, leading the way for central Africa in the management and future eradication of brucellosis.

## Supporting information

S1 Fig (TIFF)
