# Supplementary figures and images for: Scoping review of brucellosis in Cameroon: Where do we stand, and where are we going?
Source: PLoS One. 2020 Sep 28;15(9):e0239854. doi: 10.1371/journal.pone.0239854 (PMC7521690; doi:10.1371/journal.pone.0239854)

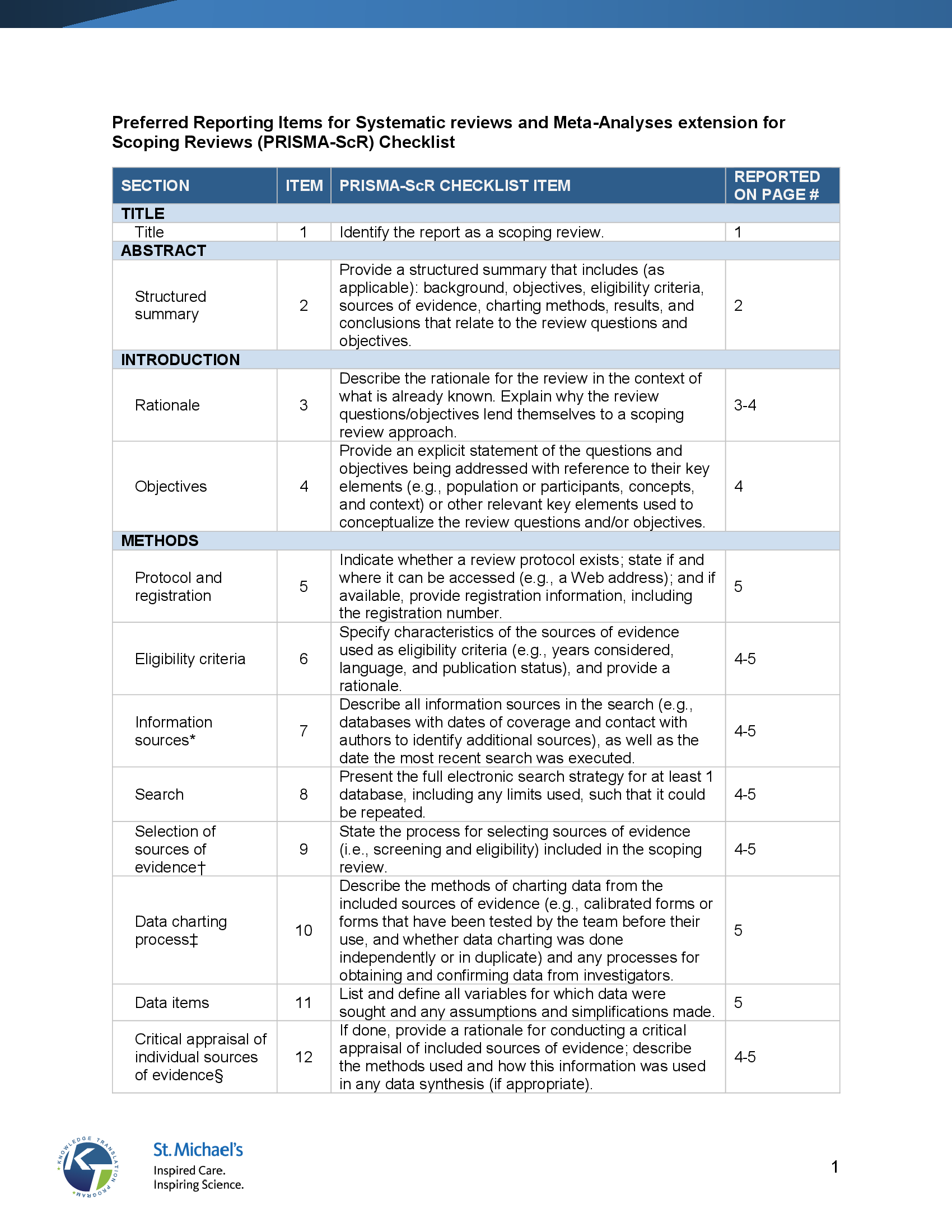

Supplement: S1 Fig — (TIFF) [file pone.0239854.s001.tiff]
